# Chaetocin: An In Vitro Bactericidal Compound That Disrupts Redox Homeostasis in *Mycobacterium tuberculosis*

**DOI:** 10.3390/microorganisms14102351

**Published:** 2026-10-14

**Authors:** Jinghua Shi, Qiyue Jia, Yangxue Ye, Bin Wang, Zimo Wang, Deqian Ji, Yu Lu

**Affiliations:** Department of Pharmacology, Beijing Key Laboratory of Drug Resistance Tuberculosis Research, Beijing Chest Hospital, Capital Medical University, Beijing Tuberculosis and Thoracic Tumor Research Institute, Beijing 101149, China

**Keywords:** chaetocin, *Mycobacterium tuberculosis*, thioredoxin reductase, redox homeostasis

## Abstract

The increasing prevalence of drug-resistant *Mycobacterium tuberculosis* underscores the urgent need for novel therapeutic strategies. We found that the natural product chaetocin exhibits in vitro bactericidal activity against drug-sensitive and drug-resistant *M. tuberculosis* strains. Biochemical and genetic analyses revealed that chaetocin exerts its antibacterial activity by disrupting bacterial thiol-redox homeostasis, with the thioredoxin reductase TrxB2 as a key mediator. Chaetocin treatment leads to a disruption of cellular thiol-redox homeostasis and accumulation of lethal reactive oxygen species. Notably, modulation of TrxB2 expression directly altered bacterial susceptibility to chaetocin, indicating that TrxB2 is involved in the response to chaetocin. These findings establish chaetocin as an in vitro active compound and a promising lead for further investigation targeting bacterial redox homeostasis and provide a promising foundation for the development of a new class of anti-tuberculosis agents.

## 1. Introduction

According to the latest World Health Organization (WHO) Global Tuberculosis Report (2025), tuberculosis remains a serious global public health threat. In 2024, there were an estimated 10.7 million new tuberculosis cases worldwide, resulting in over 1.2 million deaths [1]. Although data indicate a gradual decline in tuberculosis incidence and mortality, the pace of this progress is far from sufficient to meet the ambitious targets of the “End Tuberculosis Strategy.” Critically, nearly one-third of patients with drug-resistant tuberculosis still lack access to effective novel treatment regimens [2,3,4]. These most recent figures underscore the profound severity of the global tuberculosis burden and highlight the extreme urgency for developing innovative therapeutic agents with novel mechanisms of action, capable of tackling both drug-resistant strains and persistent bacterial populations [5].

**Figure 1:**
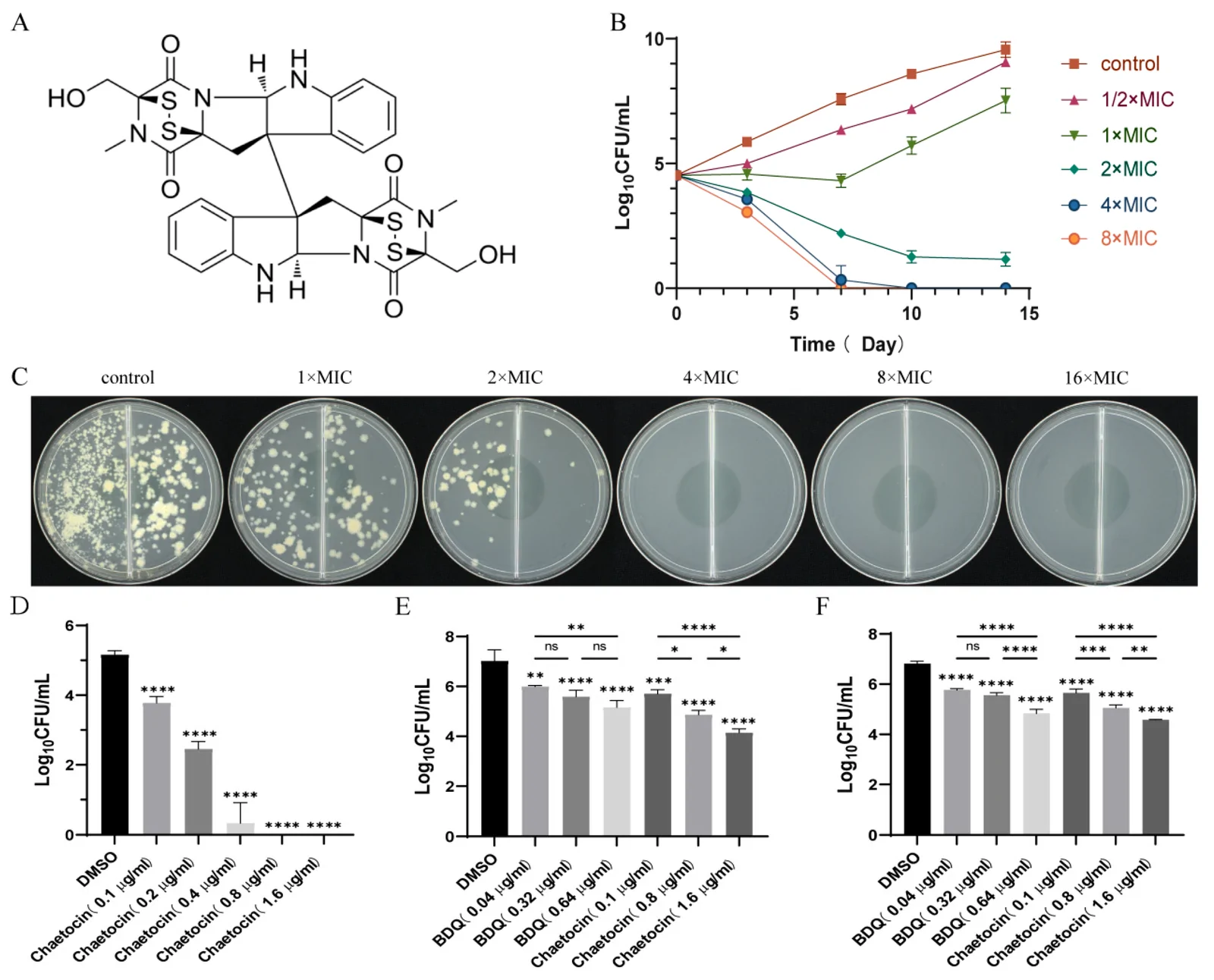
Chaetocin displays potent activity against replicating and nonreplicating *M. tuberculosis*: (**A**) Structure of chaetocin. (**B**) Time–kill kinetics of chaetocin in 7H9 medium. Presentation of MBC results (**C**) and supporting CFU enumeration (**D**) for chaetocin against *M. tuberculosis* H37Rv. Chaetocin activity against nonreplicating *M. tuberculosis* H37Rv. (**E**) Nonreplicating bacteria were starved in PBS for 2 weeks before treatment. Replicating cultures were grown in 7H9 medium. Cultures were treated for 1 week with the drug. (**F**) Nonreplicating bacteria were cultured in sealed tubes with the addition of methylene blue indicator for one week, followed by drug treatment for another week; * *p* < 0.05, ** *p* < 0.01, *** *p* < 0.001, **** *p* < 0.0001 compared with DMSO; ns, not significant (*p* ≥ 0.05). Data are presented as mean ± SD from three independent experiments. Statistical significance was determined by one-way ANOVA followed by Tukey’s post hoc test.

Chaetocin is a natural product derived from fungi of the Chaetomium genus, featuring a unique bridged epidithiodiketopiperazine (ETP) scaffold. This structure is rich in disulfide bonds, which confer high thiol reactivity, enabling it to covalently bind to the sulfhydryl (-SH) groups at the enzyme active site and thereby irreversibly inhibit the function of target enzymes (Figure 1A) [6,7]. In this study, through screening a small-molecule compound library, we discovered that chaetocin exhibits potent bactericidal activity against *M. tuberculosis*, including non-replicating bacterial populations. However, its precise molecular target and mechanism of action in exerting anti-tuberculosis effects remain unelucidated and necessitate further in-depth investigation.

Notably, antimicrobial agents with similarly thiol-reactive structures, such as the disulfide-containing compounds thiolutin and allicin, exert their effects via distinct mechanisms that involve bacterial redox systems. Thiolutin is activated upon reduction within *actinomycete* cells, leading to zinc chelation and metabolic disruption [8]. Allicin modifies protein thiols in *Staphylococcus aureus* through S-thioallylation, inducing widespread oxidative stress [9]. These precedents suggest that the potent anti-tuberculosis activity of chaetocin is likely rooted in its unique, thiol-reactive chemical scaffold. This structural characteristic distinguishes it from the scaffolds of current first-line anti-tuberculosis drugs, therefore indicating that chaetocin may possess a novel mechanism of action worthy of further in-depth investigation.

In this study, we identified the natural product chaetocin as a potent agent against *M. tuberculosis*. Through a series of phenotypic screenings, biochemical analyses, and genetic validations, we discovered that chaetocin potently disrupts bacterial intracellular redox homeostasis, leading to a massive accumulation of reactive oxygen species and subsequent irreversible oxidative damage and bacterial death. Our work reveals that chaetocin disrupts bacterial redox homeostasis, with TrxB2 as a key mediator, providing an important lead compound and theoretical foundation for the development of a new generation of anti-tuberculosis drugs aiming to eradicate persister cells.

## 2. Materials and Methods

**Mycobacterial strain.** The *M. tuberculosis* H37Rv strain (ATCC 27294) was grown in 7H9 broth supplemented with 10% Middlebrook acid–albumin–dextrose–catalase (OADC) Enrichment (BD Difco, Franklin Lakes, NJ, USA), 0.2% glycerol, and 0.05% Tween 80. Log-phase cultures incubated at 37 °C with 5% CO_2_ were used in all studies.

**Strain construction.** The *trxB2*-overexpressing strain (TrxB2-OE) was constructed as follows: The *trxB2* gene (*Rv3913*) was amplified by PCR using genomic DNA from *M. tuberculosis* H37Rv as a template. The amplified fragment was cloned into the expression vector pMV261 to generate the recombinant plasmid pMV261-*trxB2*. The recombinant plasmids were first transformed into *E. coli* DH5α, verified by colony PCR and Sanger sequencing, and then electroporated into wild-type H37Rv. Transformants were selected on kanamycin-containing plates. Overexpression was verified by qRT-PCR and Western blotting.

**Antimicrobial agents.** Anti-Bacterial Compound Library and chaetocin were purchased from TargetMol. Isoniazid (INH), rifampicin (RIP), bedaquiline (BDQ), clofazimine (CFZ), moxifloxacin (MXF), linezolid (LZD), ethambutol (EMB), levofloxacin (LVX), and pretomanid (PA-824) were purchased from Sigma-Aldrich.

**Compound library screening.** The library used was the commercially available Natural Product Library (Selleck, Cat. No. L1400), containing 3667 compounds. Primary screening was performed against *M. tuberculosis* H37Rv at a single concentration, followed by MIC determination for active hits.

**Clinical isolate susceptibility testing.** Clinical *M. tuberculosis* isolates were obtained from Beijing Chest Hospital. Drug susceptibility testing was performed using standard proportion methods and MIC assays for INH, RIF, MFX, BDQ, and CFZ. Resistance phenotypes are summarized in Table S1.

**Minimum inhibitory concentration determination.** The test compounds were subjected to two-fold serial dilutions in 96-well plates. Each well received 100 μL of a bacterial suspension, resulting in a final concentration of 1 × 10^5^ CFU/mL. Plates were incubated at 37 °C under 5% CO_2_ for 7 days. Following incubation, alamarBlue reagent containing 20% Tween 80 was added to each well, and the plates were incubated for an additional 24 h. Fluorescence intensity was then measured using a microplate reader at excitation/emission wavelengths of 530/590 nm.

**Minimum bactericidal concentration.** *M. tuberculosis* H37Rv was treated at a final concentration of 1 × 10^5^ CFU/mL. Bacterial suspensions were sampled on days 0 and 7 of incubation, appropriately diluted, and plated onto 7H10 solid agar. After incubation, colony-forming units (CFUs) were enumerated. The bactericidal activity was assessed by comparing the changes in Log_10_CFU/mL across different drug concentrations, and the minimum bactericidal concentration (MBC) was determined accordingly.

**Checkboard experiment in vitro.** Based on the MIC values, two drugs were subjected to two-dimensional continuous twofold serial dilution in a 96-well plate: one drug was diluted horizontally (rows B to H), and the other was diluted vertically (columns 2 to 8). Each well received 100 µL of *M. tuberculosis* H37Rv culture in the logarithmic growth phase at a concentration of 1 × 10^5^ CFU/mL. The alamarBlue assay was similarly used to assess the combined inhibitory concentrations.

**Time–kill kinetics assay.** Bacterial cultures (2× 10^5^ CFU/mL) were treated with chaetocin at concentrations ranging from 1/2× MIC to 8× MIC. At days 0, 3, 7, 10, and 14, 10-fold serial dilutions of the cultures were plated on 7H10 solid agar. After 3 to 4 weeks of incubation, CFUs were enumerated, and bactericidal activity was analyzed by plotting log_10_ CFU/mL. According to the fluorescence and color changes in the incubation medium, the combination MICs were determined based on the minimal drug concentrations without obvious bacterial growth. The fractional inhibitory concentration (FIC) index was defined as [MIC_A_ in combination]/[MIC_A_ alone] + [MIC_B_ in combination]/[MIC_B_ alone]. MIC_A_ alone and MIC_B_ alone are the MICs obtained when each drug was tested alone, and MIC_A_ in combination and MIC_B_ in combination are the concentrations of each compound at the lowest effective combination. The FIC was calculated to classify the interactions of three types: synergy (FIC index ≤ 0.5), indifference (0.5 < FIC index ≤ 4), and antagonism (FIC index > 4). The checkerboard assays were performed in three independent experiments.

**Nonreplicating *M. tuberculosis* screening.** The H37Rv culture was centrifuged, resuspended in PBS, and adjusted to a concentration of 5 × 10^6^ CFU/mL. It was then incubated statically at 37 °C under 5% CO_2_ for approximately 2 weeks. This culture was transferred to a 48-well plate and treated with drug solutions. After 7 days of further incubation, samples were plated onto 7H10 solid medium, and bactericidal activity against persister cells was assessed via colony-forming unit (CFU) enumeration.

The bacterial culture was inoculated into sealed glass tubes containing Dubos medium supplemented with methylene blue indicator, followed by slow oscillatory incubation at 37 °C. Once the methylene blue had completely faded to colorless (typically after about 7 days), a stable hypoxic environment was established. Drug solutions were then injected into the tubes using sterile syringes. After an additional 7 days of incubation, CFU counting was performed to evaluate drug activity.

**RNA sequencing and analysis.** RNA sequencing and analysis. H37Rv cultures were grown in 7H9 medium to mid-log phase (OD_600_ 0.6–0.8) and treated with DMSO, 4× MIC, or 8× MIC chaetocin for 24 h. Three independent biological replicates were used. Total RNA was purified using the Qiagen^®^ RNA Purification Kit (Column) (Shanghai Biotechnology Corporation, Shanghai, China) according to the manufacturer’s instructions. RNA integrity was assessed using an Agilent Bioanalyzer 5300 (Agilent Technologies, Santa Clara, CA, USA). Sequencing was performed on the Illumina NovaSeq 6000 platform (Illumina, USA), with approximately 20 million paired-end reads per sample. Raw reads were quality-filtered and mapped to the *M. tuberculosis* H37Rv reference genome (NC_000962.3) using Bowtie2 (version 2.2.0.5), with a mapping rate >90%, and gene counts were generated with HTSeq. Normalization was performed using the trimmed mean of M-values (TMM) method implemented in edgeR (version 4.6.2). Differential expression analysis was conducted using the negative binomial generalized linear model with the exactTest function in edgeR. *p*-Values were adjusted for multiple testing using the Benjamini–Hochberg procedure to control the false discovery rate (FDR). Differentially expressed genes were identified with FDR < 0.05 and |log_2_ fold change| > 1. GO and KEGG pathway enrichment analyses were performed in R (version 3.2.1), and results were visualized using ggplot2.

**ROS assays.** *M. tuberculosis* H37Rv in the logarithmic growth phase was treated with chaetocin for 24 h. Subsequently, intracellular reactive oxygen species (ROS) levels were measured using a commercial ROS detection kit (BestBio, BB-461113) according to the manufacturer’s instructions.

**Determination of lipid peroxidation level.** Mycobacterial cultures were treated with chaetocin and a positive control drug (1× LpoUp, included in the kit) for 24 h. Lipid peroxidation was measured using a Lipid Peroxidation Detection Kit (Beyotime, BODIPY 581/591 C11).

**Free thiol assay.** Mycobacterial cultures were treated with the drug for 24 h. Bacterial pellets were collected by centrifugation and washed with PBS. The intracellular free thiol content was then measured using a Thiol Detection Assay Kit (Cayman, 700340) according to the manufacturer’s protocol.

**Cellular concentrations of cofactors.** Mycobacterial cultures were treated with the drug for 24 h. Subsequently, the intracellular levels of NADP^+^ and NADPH were measured using a NADP^+^/NADPH Ratio Assay Kit (Amplite, 15264) following the manufacturer’s protocol.

**Membrane permeability and integrity assay.** Bacteria in the logarithmic growth phase were treated with different concentrations of the drug for 24 h. The bacterial suspension was then co-incubated with 10 μM NPN and 20 μM PI in the dark at 37 °C with continuous shaking for 1 h. Fluorescence intensity was measured using a multifunctional microplate reader (NPN: excitation/emission wavelengths 350/420 nm; PI: excitation wavelength 535 nm, emission wavelength 615 nm).

**ATP assay.** ATP levels were measured using the ATP Assay Kit (Beyotime, S0026) following a 24 h treatment of *M. tuberculosis* cultures with the drug.

**Protein expression, purification, and activity assays.** The *trxB2* (*Rv3913*) and *trxC* (*Rv3914*) genes of *M. tuberculosis* were cloned into the pET28a expression vector, and the corresponding proteins were purified by Ni-NTA affinity chromatography. TrxB2 activity was measured at 37 °C in black clear-bottom 96-well plates with a total reaction volume of 200 μL. The standard reaction mixture contained 25 nM TrxB2, 100 μM DTNB, 100 μM NADPH, and 50 nM TrxC, prepared in 50 mM Hepes (pH 7.5) containing 2 mM EDTA. Absorbance at 412 nm was monitored using a microplate reader to indicate enzymatic activity.

**Fluorescence quenching assay.** The protein stock solution was diluted with 100 mM dipotassium hydrogen phosphate protein concentration exchange buffer, and its fluorescence intensity was measured at wavelengths of 330–500 nm. A suitable protein concentration was selected as the fixed concentration. To 96 μL of the protein solution, 4 μL of the drug at varying concentrations was added, followed by measurement of its fluorescence intensity.

**Surface plasmon resonance.** The protein was diluted to 22 μg/mL using Acetate 4.0 buffer and then captured on channel 2 of a CM5 chip with a capture level of 12740 RU. Subsequently, the analyte was subjected to a 2-fold serial dilution using 1× PBS-P containing 2% DMSO to generate 12 concentration gradients: 15000 nM, 7500 nM, 3750 nM, 1875 nM, 937.5 nM, 468.75 nM, 234.37 nM, 117.19 nM, 58.59 nM, 29.30 nM, 14.65 nM, and 7.32 nM.

**Statistical analysis.** Data are presented as mean ± SD from three independent experiments unless otherwise noted. Comparisons were performed using one-way ANOVA followed by Tukey’s post hoc test. A *p* value < 0.05 was considered statistically significant.

## 3. Results

### 3.1. Chaetocin Exhibits Potent Bactericidal Activity Against M. tuberculosis

In a search for novel anti-tuberculosis lead compounds, we screened the Natural Product Library (Selleck, Cat. No. L1400, containing 3667 compounds) against *M. tuberculosis* H37Rv under replicating conditions at a single concentration, followed by MIC determination for active hits. This effort identified the natural product chaetocin as a hit compound with significant inhibitory activity against *M. tuberculosis*. Chaetocin demonstrated a remarkably low minimum inhibitory concentration (MIC) of 0.1 μg/mL against the strain *M. tuberculosis* H37Rv. Importantly, this potent activity was maintained across a panel of clinical isolates, including strains sensitive and resistant to first- and second-line drugs such as isoniazid, rifampicin, and fluoroquinolones, with no significant increase in MIC values observed (Table 1), indicating a lack of cross-resistance. To further characterize these clinical isolates, we determined their MICs against INH, RIF, MFX, BDQ, and CFZ and classified them as susceptible or resistant using standard MIC breakpoints (Supplementary Table S1).

We subsequently evaluated the interaction between chaetocin and standard tuberculosis drugs using a checkerboard assay. Chaetocin demonstrated values consistent with the predefined threshold for synergistic effect when combined with clofazimine (CFZ) and pretomanid (PA-824). Indifferent interactions were observed when chaetocin was combined with isoniazid (INH), rifampicin (RIF), linezolid (LZD), levofloxacin (LVX), bedaquiline (BDQ), or ethambutol (EMB). Notably, no antagonistic interactions were detected with any of the tested drugs (Table 2).

To characterize the killing dynamics of chaetocin, time–kill kinetics analysis was performed against *M. tuberculosis* H37Rv. The results revealed that the killing effect was both concentration- and time-dependent. Treatment with 0.4 μg/mL (4× MIC) for 7 days reduced the bacterial culture to near sterility (Figure 1B). The bactericidal potency was further quantified by determining the minimum bactericidal concentration (MBC), which was found to be 0.4 μg/mL (4× MIC). Following 7 days of exposure to this concentration, an approximately 4.83-log_10_ reduction in bacterial load was observed, confirming its robust killing kinetics (Figure 1C,D).

Critically, to evaluate its activity against non-replicating, persistent bacteria—a major challenge in tuberculosis therapy—we tested chaetocin in established models of dormancy. In a nutrient-starvation model, treatment with 1.6 μg/mL resulted in an approximately 2.96-log_10_ reduction in bacterial load (Figure 1E). Similarly, in a hypoxia model mimicking the granulomatous environment, we observed an approximately 2.23-log_10_ reduction in bacterial load at 1.6 μg/mL (Figure 1F). These results demonstrate that chaetocin retains potent bactericidal activity against phenotypically drug-tolerant persister populations.

### 3.2. Chaetocin Disrupts Essential Pathways for the Growth and Development of M. tuberculosis

**Figure 2:**
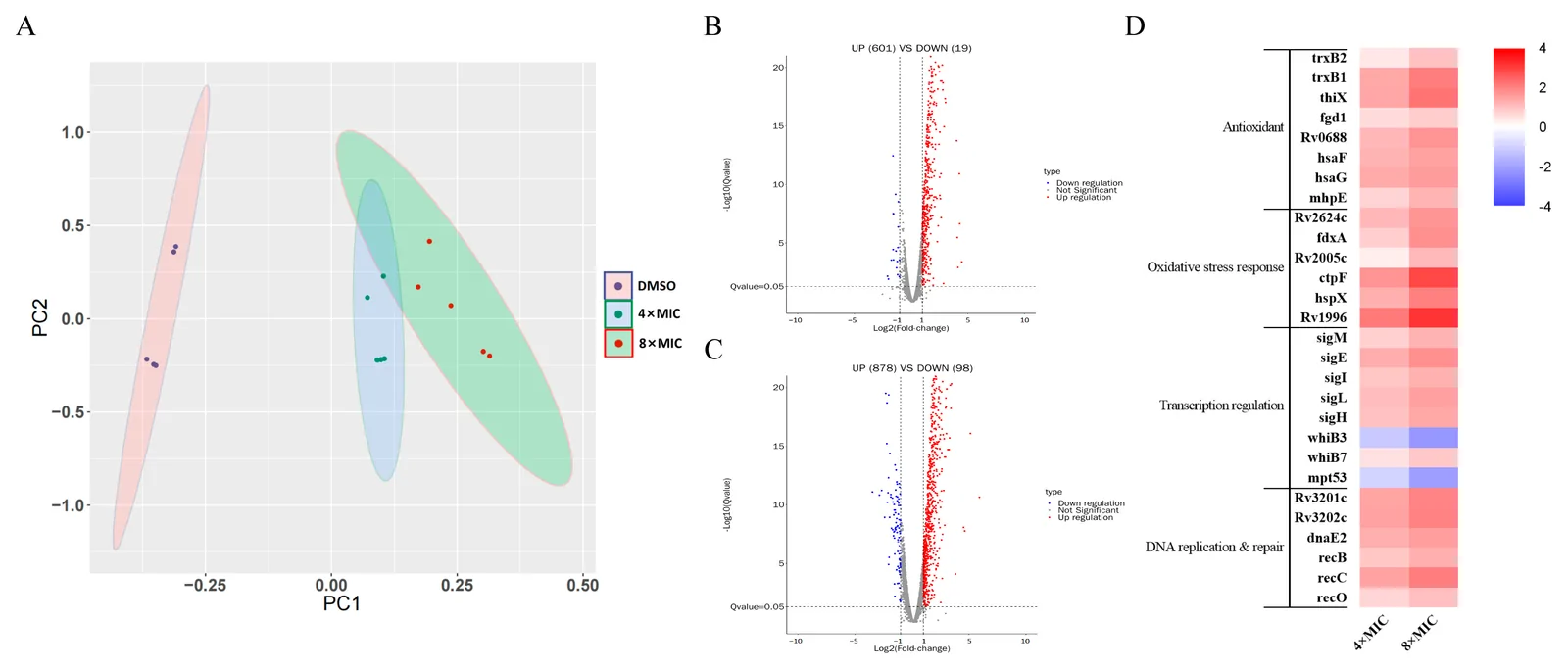
Chaetocin disrupts essential pathways for the growth and development of *M. tuberculosis*: (**A**) Scatter plots showing the PCA score plots of strains treated with 4× MIC or 8× MIC chaetocin versus the DMSO control group. Volcano plots of differentially expressed genes after 24 h treatment with chaetocin at 4× MIC (**B**) and 8× MIC (**C**). (**D**) Heatmap showing differentially expressed genes within the enriched pathways after 24 h treatment with chaetocin at 4× MIC and 8× MIC. Data are presented as log_2_ fold changes relative to the DMSO control group from the same batch.

Given its significant activity against clinically drug-resistant strains, along with its structural characteristics that are distinctly different from existing drugs, we hypothesized that chaetocin might possess a novel mechanism of action distinct from current anti-tuberculosis agents and therefore initiated subsequent investigations into its mechanism of action. This study aimed to investigate the pathways affected by chaetocin in *M. tuberculosis* and analyze the associated transcriptomic changes. Transcriptomic analysis was performed on mycobacteria treated with different concentrations of chaetocin, using DMSO-treated groups from the same batch as controls. The unit variance between the control and treated groups was determined by providing all the data points with intensity to multi-group inputs, and score principal component analysis (PCA) plots were generated (Figure 2A). Therefore, the PCA scores revealed that transcriptomics of the chaetocin-treated and untreated samples differed from each other at all tested concentrations. Subsequently, we treated the bacterial strains with chaetocin at concentrations of 4× MIC and 8× MIC for 24 h and generated volcano plots to illustrate the differentially expressed genes in each comparison (Figure 2B,C). Functional enrichment analysis indicated that these genes were primarily involved in pathways related to oxidative stress response, DNA repair, transcription, translation, and post-translational modifications (Figure 2D).

Notably, genes associated with antioxidant defense and oxidative stress responses showed significant differential expression after chaetocin treatment, with the expression levels of core antioxidant enzyme genes—including *trxB2*, *trxB1*, and *fgd1*—being markedly upregulated. These genes encode thioredoxin reductase and its cofactor-generating enzymes, which constitute the primary defense line against oxidative stress in bacteria [10,11,12]. Their upregulation suggests that high concentrations of chaetocin directly suppress or disrupt the core antioxidant defense system of *M. tuberculosis*, prompting the bacterium to upregulate oxidative stress-responsive genes in an attempt to restore its “detoxification” capacity. Bacteria perceive stress signals induced by oxidative stress, thereby activating the expression of genes such as *fdxA*, *Rv1996*, and *ctpF* to help maintain the intracellular reducing environment and counteract oxidative stress [13,14,15]. The increased expression of genes such as *sigH*, *sigE,* and *sigM* suggests that the bacteria perceive changes in redox status and initiate large-scale gene expression reprogramming to cope with the stress. Genes such as *recB*, *recC*, and *recO* are activated following DNA damage caused by the burst of reactive oxygen species, functioning to repair and maintain genomic integrity [16,17,18]. This suggests that chaetocin may induce a stress response by disrupting the intracellular redox homeostasis of *M. tuberculosis*.

### 3.3. Chaetocin Treatment Depletes Thiols and Impairs the Defense Against Oxidative Stress in M. tuberculosis

**Figure 3:**
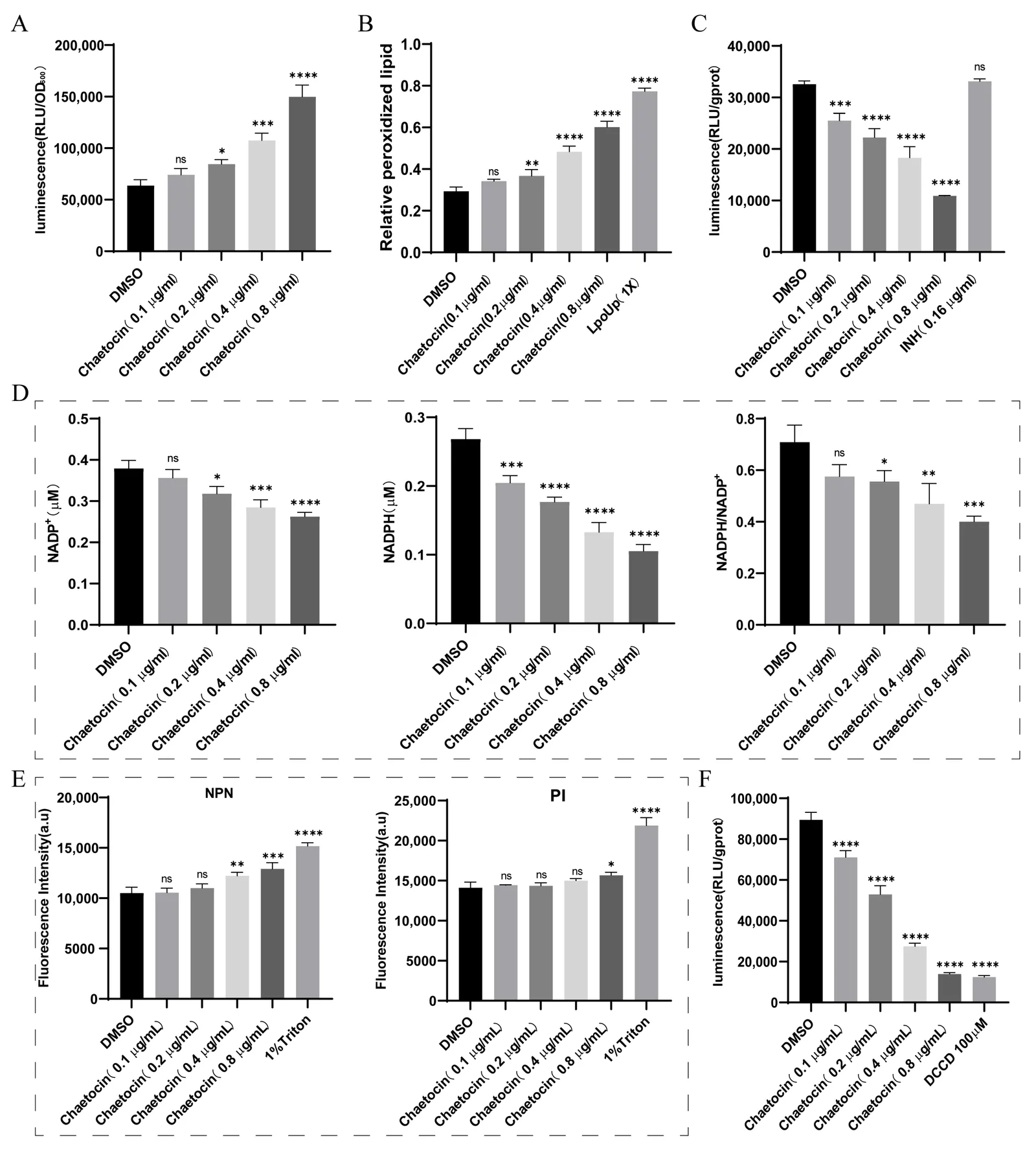
Chaetocin depletes intracellular thiols, disrupts redox homeostasis, and induces oxidative stress in *M. tuberculosis*. Effects of 24-h chaetocin treatment at varying concentrations on intracellular ROS (**A**), lipid peroxidation (**B**), thiol homeostasis (**C**), NADPH/NADP^+^ levels (**D**), membrane permeability (**E**), and ATP (**F**) in replicating *M. tuberculosis*; * *p* < 0.05, ** *p* < 0.01, *** *p* < 0.001, **** *p* < 0.0001 compared with DMSO; ns, not significant (*p* ≥ 0.05). Data are presented as mean ± SD from three independent experiments. Statistical significance was determined by one-way ANOVA followed by Tukey’s post hoc test.

Based on the above transcriptomic analysis results—which revealed that chaetocin treatment induced a broad upregulation of genes related to antioxidant defense and oxidative stress response in *M. tuberculosis*, particularly the significant upregulation of core reductive system genes such as thioredoxin reductase TrxB2—we hypothesized that chaetocin likely disrupts the intracellular redox homeostasis of the bacteria, thereby triggering lethal oxidative stress. To test this hypothesis, we first measured the levels of reactive oxygen species (ROS) (Figure 3A) and lipid peroxidation (Figure 3B) in chaetocin-treated bacteria. The results showed that as the drug concentration increased, ROS accumulation increased significantly, and lipid peroxidation levels also rose markedly, indicating that the bacteria were under severe oxidative stress [19].

Subsequently, we determined the levels of free thiols (Figure 3C) and NADPH (Figure 3D) to assess whether the redox homeostasis was impaired. The results revealed that after chaetocin treatment, free thiols in the bacteria decreased significantly, and NADPH levels gradually declined with increasing drug concentration, leading to an imbalance in the NADPH/NADP^+^ ratio and severely compromising the bacteria’s ability to cope with oxidative stress.

NPN is a hydrophobic probe that fluoresces upon partitioning into damaged outer membranes, while PI enters only cells with disrupted cytoplasmic membranes and intercalates into DNA [20]. To further investigate the structural damage caused by the drug, we measured membrane permeability-related indicators, including NPN (1-N-phenylnaphthylamine) and PI (propidium iodide) fluorescence intensity (Figure 3E), and assessed intracellular ATP levels (Figure 3F). The results indicated that chaetocin treatment increased membrane permeability, although membrane integrity remained largely intact at low concentrations. Meanwhile, intracellular ATP levels decreased significantly, suggesting severe inhibition of energy metabolism.

In summary, chaetocin disrupts the redox homeostasis of *M. tuberculosis*, leading to the collapse of its antioxidant system and energy metabolism exhaustion, ultimately resulting in severe bacterial damage.

### 3.4. Chaetocin Affects TrxB2 Enzymatic Activity In Vitro

The thioredoxin reductase of *M. tuberculosis* (TrxB2) belongs to the family of low-molecular-weight bacterial disulfide-dependent thioredoxin reductases, sharing less than 30% homology with human thioredoxin reductase (TrxR). Human TrxR originates from an independent evolutionary lineage, and its catalytic mechanism differs significantly from that of the bacterial enzyme [21]. Chaetocin is rich in disulfide bonds, and TrxB2 is a key bacterial enzyme responsible for transferring reducing equivalents from NADPH to substrates. Experimental findings show that after chaetocin treatment, the expression of thioredoxin system-related genes (especially trxB2) is upregulated, while intracellular free thiol levels are significantly decreased. Based on these observations, we hypothesize that chaetocin may affect the activity of the TrxB2 protein.

**Figure 4:**
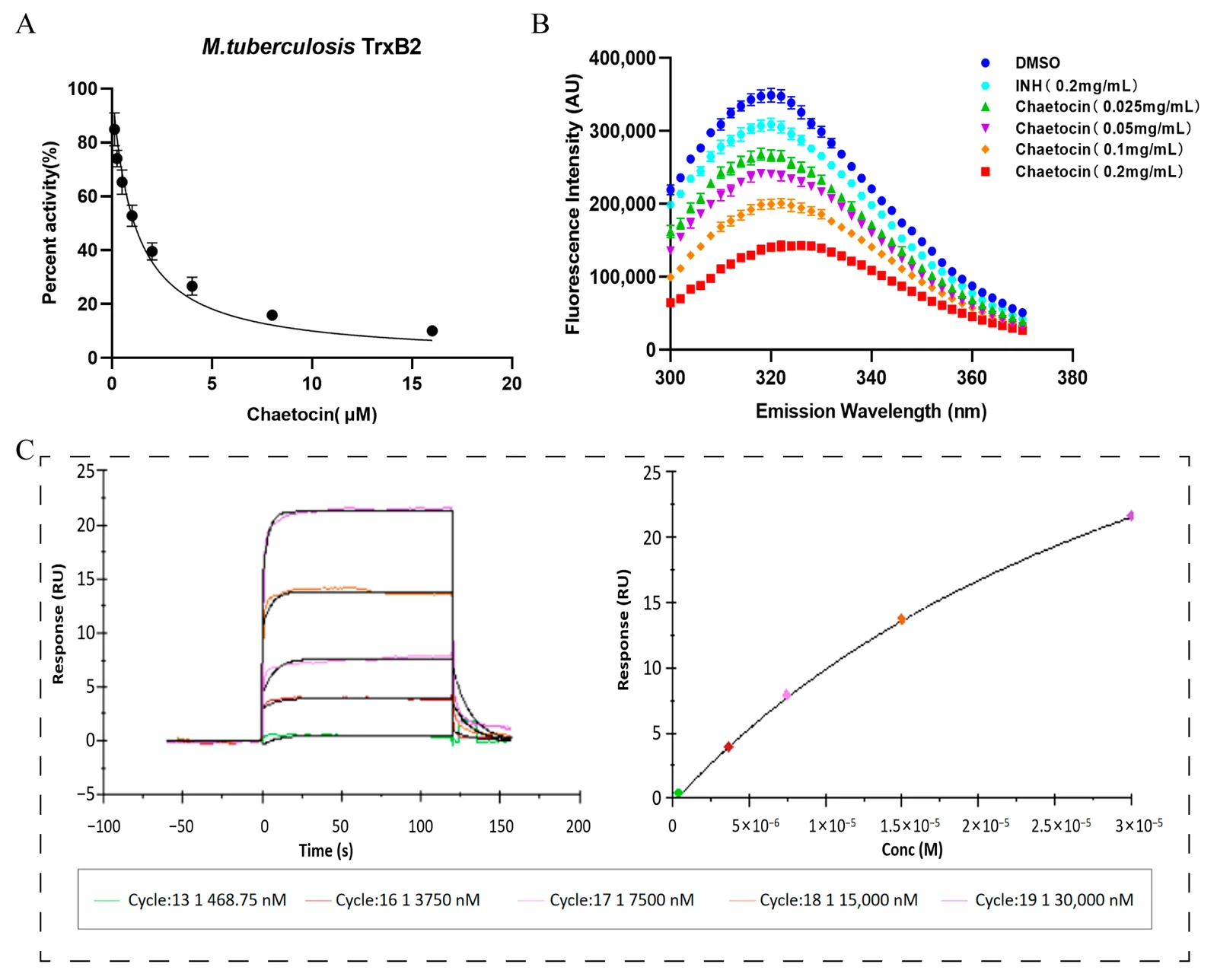
Chaetocin inhibits bacterial TrxB2: (**A**) Purified recombinant *M. tuberculosis* TrxB2 was preincubated with NADPH and DTNB with or without chaetocin for 15 min. Reactions were initiated by the addition of TrxC. Shown is a dose–response plot for the initial rate of TrxB2 activity in the presence or absence of chaetocin. (**B**) Fluorescence intensity of the TrxR protein after binding to chaetocin. (**C**) The CM5 chip immobilized thioredoxin reductase as the ligand. From 0 to 120 s, thioredoxin reductase bound to chaetocin at concentrations of 30,000 nM, 15,000 nM, 7500 nM, 3750 nM, and 468.75 nM, respectively, followed by dissociation between 120 and 170 s.

To test this hypothesis, we cloned the TrxB2 gene of *M. tuberculosis* and its substrate thioredoxin (trxC) into an expression vector containing an N-terminal His-tag. After expression in *E. coli*, the proteins were purified using nickel-charged agarose resin. A thioredoxin reductase activity assay was performed in the presence of TrxC [22]. Enzyme activity was measured using a reaction that regenerates oxidized TrxC through a rapid intramolecular disulfide exchange with 5,5′-dithiobis-(2-nitrobenzoic acid) (DTNB) [23]. In this assay, the reaction progress was monitored by measuring the absorbance of the released TNB chromophore at 412 nm. The results showed that when pre-incubated with TrxR and NADPH, chaetocin strongly inhibited the TrxB2 enzyme of *M. tuberculosis* in a dose-dependent manner, with a half-maximal inhibitory concentration (IC_50_) of 1.12 ± 0.6 µM for TrxB2 (Figure 4A).

We then employed fluorescence quenching and surface plasmon resonance (SPR) techniques to examine whether chaetocin interacts with the TrxB2 protein. Fluorescence is a photoluminescence phenomenon in which molecules absorb radiation and emit light at a wavelength close to that of the incident radiation [24]. The intrinsic fluorescence of proteins primarily originates from tryptophan (Trp), tyrosine (Tyr), and phenylalanine (Phe) residues [25,26]. Fluorescence quenching results showed that after adding equal volumes of chaetocin at various concentrations, the fluorescence intensity of TrxB2 gradually decreased in a concentration-dependent manner. Moreover, at equivalent concentrations, chaetocin significantly reduced the fluorescence intensity of TrxB2 compared to isoniazid. (Figure 4B).

In SPR experiments designed to measure small molecule-protein interactions, the protein is immobilized on the biosensor surface via amine coupling, while the small molecule flows over the sensor chip. The interaction between the small molecule and the protein is monitored through subsequent changes in the refractive index and quantified in resonance units [27,28]. The binding between the TrxB2 protein and chaetocin is characterized by rapid association and dissociation. For such fast-binding monomolecular kinetic processes, an appropriate binding model can be applied for analysis. The results demonstrated an interaction between the TrxB2 protein and chaetocin, with an affinity constant (KD) of 12 µM (Figure 4C).

### 3.5. Overexpression of the trxB2 Gene Helps Resist the Disruption of Intracellular Redox Homeostasis by Chaetocin

To further investigate whether TrxB2 is involved in the chaetocin response, we constructed a *trxB2*-overexpressing strain and evaluated the effect of TrxB2 on the antitubercular activity of chaetocin in bacteria. First, we examined the impact of TrxB2 overexpression on the minimum inhibitory concentration (MIC) of chaetocin. The results showed that TrxB2 overexpression increased the MIC of chaetocin by 8-fold, whereas the MIC values of the control drugs isoniazid and rifampin remained unchanged (Table 3).

**Figure 5:**
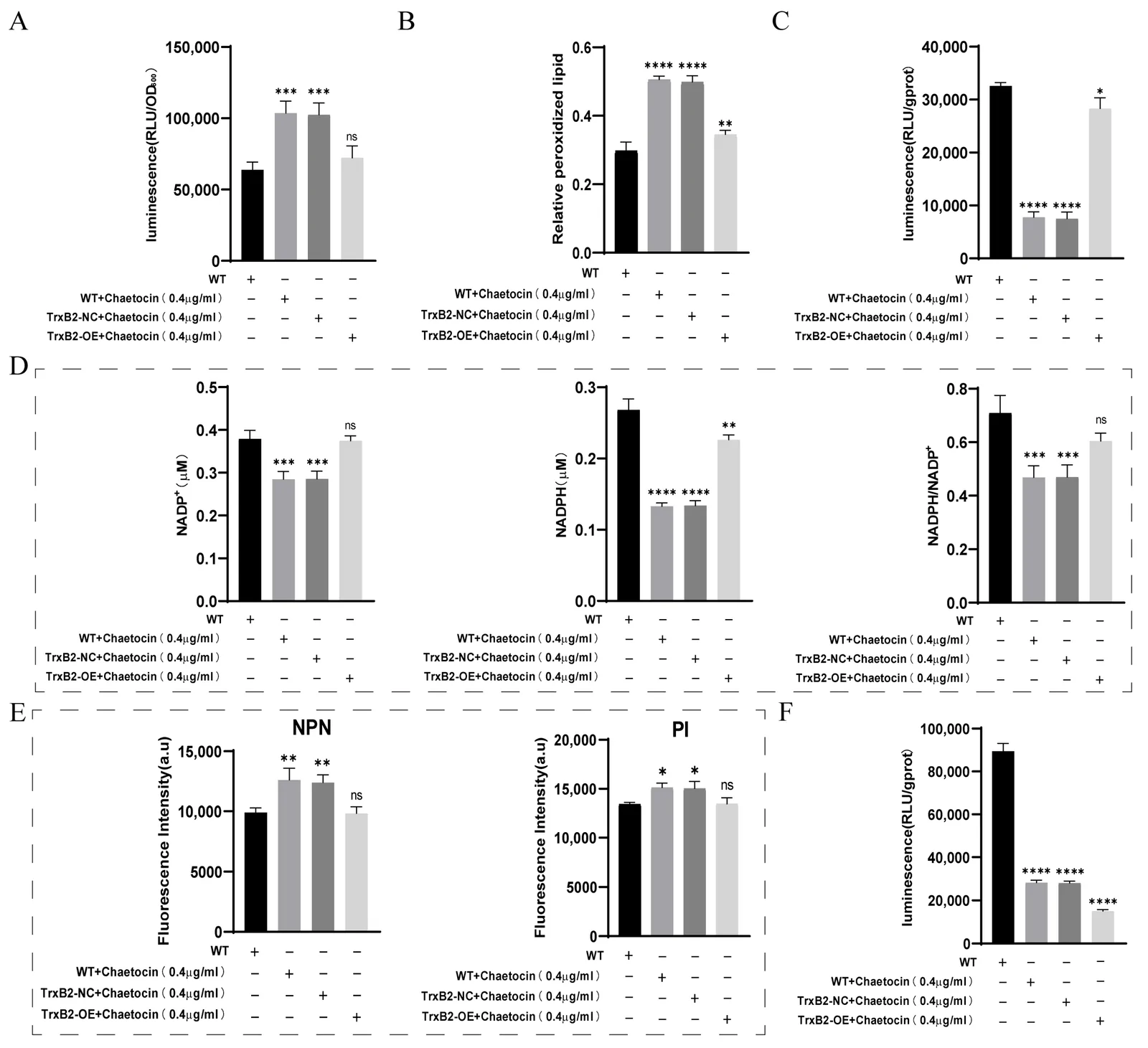
The effects of chaetocin on intracellular ROS (**A**), lipid peroxidation (**B**), thiol homeostasis (**C**), NADPH/NADP^+^ levels (**D**), membrane permeability (**E**), and ATP (**F**) were measured in different *M. tuberculosis* strains, including H37Rv, TrxB2-NC (pMV261), and a *trxB2*-overexpressing strain (pMV261-TrxB2); * *p* < 0.05, ** *p* < 0.01, *** *p* < 0.001, **** *p* < 0.0001 compared with DMSO; ns, not significant (*p* ≥ 0.05). Data are presented as mean ± SD from three independent experiments. Statistical significance was determined by one-way ANOVA followed by Tukey’s post hoc test.

Subsequently, we compared the redox homeostasis and cellular damage among the *M. tuberculosis* standard strain, empty vector-containing strain, and *trxB2*-overexpressing strain after treatment with chaetocin. The results demonstrated that in the *trxB2*-overexpressing strain, intracellular ROS levels were significantly reduced compared with the standard strain after 24 h of chaetocin treatment (Figure 5A), and lipid peroxidation was markedly alleviated (Figure 5B), indicating a mitigation of oxidative stress in the bacteria. Furthermore, both thiol content and NADPH levels were significantly restored in this strain (Figure 5C,D), suggesting a recovery of reductive capacity to counteract oxidative stress.

Finally, by comparing NPN, PI, and ATP levels among the strains to assess cellular damage, we found that the *trxB2*-overexpressing strain exhibited reduced membrane permeability and enhanced integrity (Figure 5E). However, ATP levels remained lower than those in the untreated standard strain (Figure 5F).

These results indicate that chaetocin disrupts the redox homeostasis of *M. tuberculosis* and that TrxB2 is a key mediator of this process. Nevertheless, the fact that *trxB2* overexpression did not fully restore ATP levels suggests that chaetocin may have additional antitubercular targets, which, together with TrxB2, may constitute a multi-target synergistic mechanism against tuberculosis.

## 4. Discussion

Tuberculosis remains one of the world’s deadliest infectious diseases, with the prevalence of drug-resistant tuberculosis and the difficulty in eradicating persister populations constituting major challenges for current treatment failure and relapse. Therefore, there is an urgent need to develop novel antibacterial agents with new mechanisms of action capable of effectively clearing persistent bacterial populations [29,30]. Through systematic screening, this study identified the natural product chaetocin as a potent anti-tuberculosis drug candidate. Its activity profile is remarkable: it exhibits low minimum inhibitory concentrations (MICs) against both drug-sensitive and drug-resistant clinical isolates of *M. tuberculosis*, with no observed cross-resistance to existing first- and second-line drugs. This lack of cross-resistance may be attributed to the unique chemical structure of chaetocin and its mechanism of action, which differs from that of currently commonly used anti-tuberculosis drugs. Cherblanc et al. (2013) pointed out that chaetocin is a non-specific inhibitor of histone lysine methyltransferases, and its activity depends on a chemically labile disulfide bridge [31]. Kulkarni et al. (2018) emphasized in their review that maintaining redox homeostasis is crucial for the survival of mycobacteria and that disrupting redox homeostasis and inducing oxidative stress with small molecules is an effective strategy for developing novel anti-tuberculosis drugs [32]. Such drugs do not rely on existing targets, providing new approaches to overcome drug-resistant strains. Particularly importantly, chaetocin remains effective at killing non-replicating bacteria in models mimicking the persistent state, such as nutrient starvation and hypoxia, directly targeting a key weakness of current chemotherapy. Furthermore, its results are consistent with the predefined threshold for synergistic effect with key second-line drugs (e.g., clofazimine and pretomanid) and indifferent interactions with other standard drugs, indicating its good compatibility and application potential within combination therapy regimens.

Given the excellent anti-tuberculosis activity exhibited by chaetocin, especially its potent activity against persisters, we further investigated its mechanism of action. Integrating transcriptomic and biochemical evidence, this study for the first time elucidates that chaetocin exerts its bactericidal effect by disrupting *M. tuberculosis* redox homeostasis. Although both *trxB2* and *trxB1* were upregulated after chaetocin treatment, they play distinct roles in the mycobacterial thioredoxin system. TrxB2 is the essential thioredoxin reductase and the core enzyme maintaining thiol-redox homeostasis. TrxB1 is a non-essential downstream thioredoxin and one of the substrates reduced by TrxB2, with functional redundancy from other thioredoxins such as TrxC [10]. We therefore focused on TrxB2 as a key mediator of the chaetocin response. The molecular mechanism lies in chaetocin’s unique disulfide-rich chemical structure, which directly affects thiol-redox pathways [33,34]. TrxB2 is a central hub for bacteria to maintain intracellular redox homeostasis; its functional inhibition leads to an imbalance in the NADPH/NADP^+^ ratio and depletion of the free thiol pool and subsequently triggers an explosive accumulation of endogenous reactive oxygen species (ROS) [10,35]. This oxidative damage cascade induces different processes, including lipid peroxidation, DNA damage, and energy metabolism (ATP) collapse, ultimately leading to bacterial death [36,37,38].

TrxB2 has been established as an essential gene in *M. tuberculosis*, playing a critical role in maintaining thiol-redox homeostasis by reducing oxidized thioredoxin (TrxC) [39]. Its essentiality and the lack of close human homologs make it an attractive, albeit underexploited, drug target. Our findings that chaetocin affects TrxB2 activity and that TrxB2 overexpression confers resistance further support the relevance of this pathway.

The findings of this study hold significant implications across multiple dimensions. From a scientific perspective, we have for the first time systematically elucidated the molecular mechanism by which the natural product chaetocin exerts its bactericidal effect through disrupting bacterial redox homeostasis. This not only deepens the understanding of the relationship between bacterial antioxidant defense systems and drug susceptibility but also provides supporting evidence for anti-tuberculosis strategies targeting this pathway. In terms of clinical translation, chaetocin exhibits potent activity against a wide range of drug-resistant strains and persister cells and shows values consistent with the predefined threshold for synergistic or indifferent interactions with existing anti-tuberculosis drugs. This offers a new candidate compound and combination therapy strategy to address the two major challenges in current tuberculosis treatment: the spread of drug resistance and the difficulty in eradicating persister populations. Furthermore, TrxB2, as a target specifically associated with prokaryotes, shows significant differences from its human homologs, laying a foundation for further investigation of novel anti-tuberculosis agents with a potential selectivity advantage, although toxicity has not been evaluated.

The elucidation of its efficacy and mechanism highlights the potential of chaetocin as a candidate compound for further investigation and reveals certain limitations of this study, pointing the way for future research. Furthermore, genetic experiments showed that although *trxB2* overexpression significantly increased bacterial resistance to chaetocin and alleviated oxidative stress, it failed to fully restore cellular ATP levels. This suggests that chaetocin’s action may not be entirely singular; besides its effect on TrxB2, there might be secondary effects on other thiol-dependent metabolic pathways, and the possibility of multi-targeting deserves further exploration. Finally, regarding the chaetocin lead compound itself, its selectivity, metabolic stability, and further structural optimization based on its active scaffold all require more systematic medicinal chemistry research to advance.

Chaetocin is also a nonspecific inhibitor of histone lysine methyltransferases, including SUV39H1, which methylates *M. tuberculosis* HupB and affects bacterial survival in host cells [31]. As this study was restricted to axenic in vitro cultures, host–pathogen interactions and host-cell toxicity were not evaluated. Chaetocin possesses potent activity against *M. tuberculosis* and merits further investigation; nevertheless, its development into an anti-tuberculosis drug still requires extensive research and optimization. Future work should include intracellular macrophage infection models, mammalian-cell cytotoxicity, selectivity, pharmacokinetics, metabolic stability, and in vivo efficacy studies.

## 5. Conclusions

**Figure 6:**
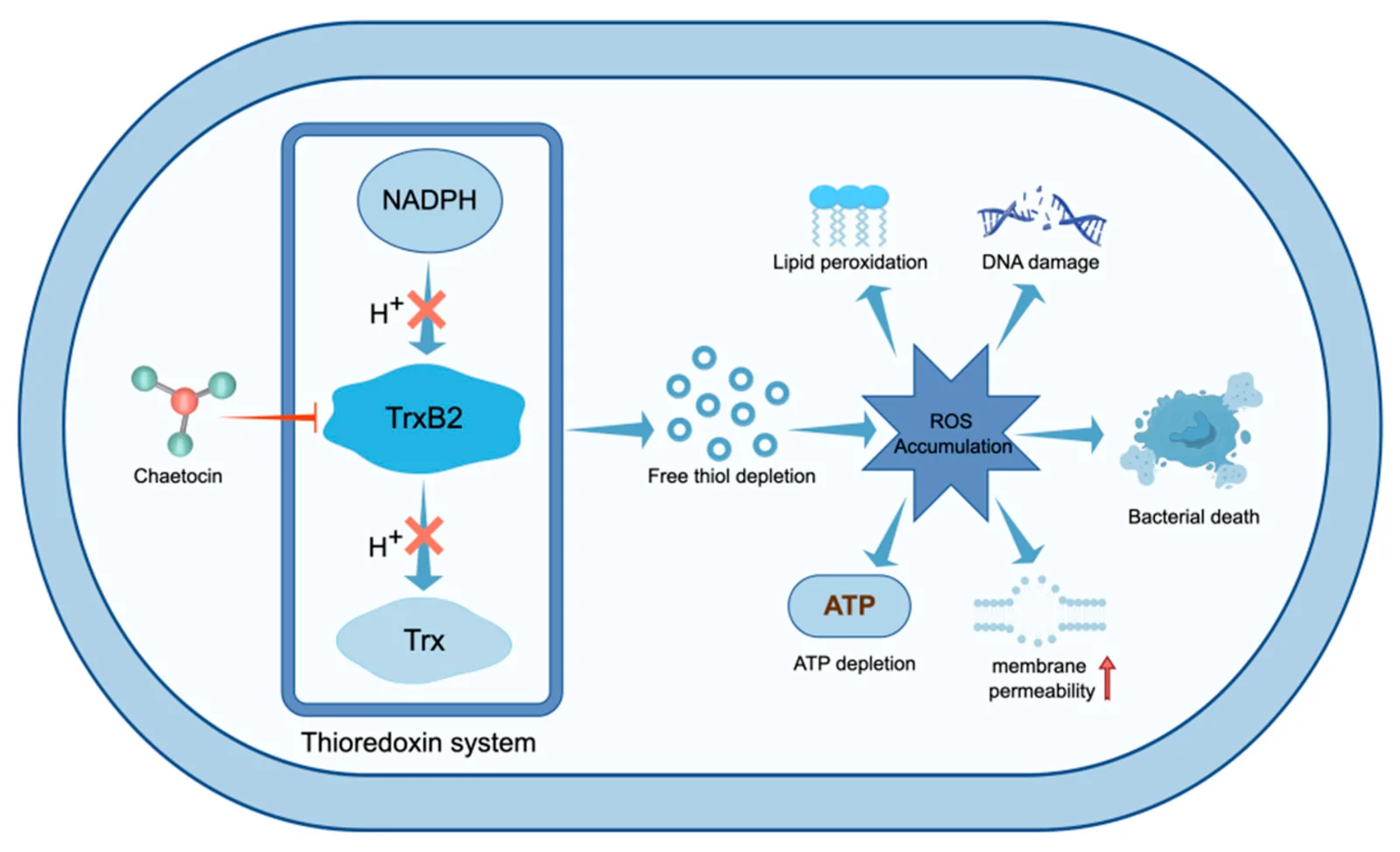
Molecular mechanism of chaetocin disrupting redox homeostasis in *M. tuberculosis* with TrxB2 as a key mediator.

This study identifies that the natural product chaetocin exhibits in vitro bactericidal activity against *M. tuberculosis*, including drug-resistant strains and persister cells. Mechanistic investigations demonstrate that chaetocin disrupts mycobacterial redox homeostasis, leading to accumulation of reactive oxygen species and bacterial death. TrxB2 is identified as a key protein involved in this process (Figure 6). This research provides a basis for further investigation of redox-targeting anti-tuberculosis strategies.

## Figures and Tables

**Table 1:** MICs of chaetocin against standard and clinically isolated susceptible and drug-resistant strains of *M. tuberculosis*.

Strains	MIC (μg/mL)					
	Chaetocin	RIF	INH	MFX	BDQ	CFZ
H37Rv	0.10	0.02	0.04	0.12	0.08	0.25
39621 ^a^	0.10	0.08	0.08	0.06	0.08	0.50
39614 ^a^	0.03	0.02	0.04	0.06	0.04	0.12
39661 ^a^	0.10	≤0.02	0.08	4.00	0.16	0.25
39655 ^a^	0.10	0.08	0.04	0.03	0.16	0.50
39773 ^a^	0.20	0.32	>2.5	0.25	0.64	2.00
39737 ^a^	0.10	0.04	>2.5	0.12	0.08	0.50
39751 ^a^	0.03	0.02	>2.5	0.06	0.02	0.06
39779 ^a^	0.05	≤0.02	>2.5	0.25	0.08	0.12
39785 ^a^	0.05	0.04	>2.5	0.12	0.04	0.12
39600 ^a^	0.10	0.32	>2.5	0.06	0.32	1.00
39682 ^a^	0.05	0.04	>2.5	0.12	0.16	1.00
39719 ^a^	0.05	0.08	>2.5	0.06	0.04	0.12
39545 ^a^	0.10	0.16	>2.5	0.03	0.04	0.12
152 ^a^	0.05	>2.5	0.02	0.06	0.01	0.12
39749 ^a^	0.20	>2.5	>2.5	0.25	0.04	0.25
39500 ^a^	0.05	>2.5	>2.5	0.06	0.04	0.12
39595 ^a^	0.10	>2.5	>2.5	2.00	0.04	0.12
39613 ^a^	0.05	>2.5	>2.5	0.50	0.08	0.25
39563 ^a^	0.10	>2.5	>2.5	>4	0.08	0.12
11 ^a^	0.10	1.25	2.50	0.25	0.01	0.03
95 ^a^	0.10	>2.5	2.50	0.03	0.01	0.12
27 ^a^	0.20	>2.5	2.50	2.00	0.01	0.25

Note: bedaquiline, BDQ; clofazimine, CFZ; isoniazid, INH; rifampicin, RIF; moxifloxacin, MFX. ^a^ Clinical strains were isolated from patients at Beijing Chest Hospital.

**Table 2:** MICs of selected anti-tuberculosis compounds against *M. tuberculosis* H37Rv and corresponding interaction profiles with chaetocin assessed by the checkerboard method.

Compound A	MIC (μg/mL)		Interaction Profile with Chaetocin	
	MIC_A_	MIC_Chaetocin_	FIC Indexes	Outcome
	Alone/Comb.	Alone/Comb.		
INH	0.04/0.02	0.1/0.0125	0.625	indifference
RIF	0.02/0.01	0.1/0.0125	0.625	indifference
LZD	0.3125/0.15625	0.1/0.025	0.75	indifference
EMB	1.25/0.625	0.1/0.05	1	indifference
LVX	0.3125/0.15625	0.1/0.025	0.75	indifference
BDQ	0.04/0.01	0.1/0.05	0.75	indifference
CFZ	0.16/0.04	0.1/0.025	0.5	synergy
PA-824	0.06/0.015	0.1/0.025	0.5	synergy

Note: isoniazid, INH; rifampicin, RIF; linezolid, LZD; ethambutol, EMB; levofloxacin, LVX; bedaquiline, BDQ; clofazimine, CFZ; pretomanid, PA-824. MIC_A_ represents the drug that is combined with chaetocin in the checkerboard method.

**Table 3:** MICs of chaetocin against standard and overexpression of the *trxB2* gene strains of *M. tuberculosis*.

Strains	MIC (μg/mL)		
	Chaetocin	RIF	INH
H37Rv	0.1	0.02	0.04
pMV261	0.1	0.02	0.04
TrxB2-OE	0.8	0.02	0.04

## Data Availability

Raw data generated from the RNA-Seq experiments of *M. tuberculosis* strains have been deposited in the NCBI Sequence Read Archive (SRA) under BioProject PRJNA1430106.

## Supplementary Materials

The following supporting information can be downloaded at: https://www.mdpi.com/article/10.3390/microorganisms14102351/s1, Table S1: Resistance phenotypes of clinical *M. tuberculosis* isolates.
