# Supplementary material for: Chaetocin: An In Vitro Bactericidal Compound That Disrupts Redox Homeostasis in Mycobacterium tuberculosis
Source: Microorganisms. 2026 Oct 14;14(10):2351. doi: 10.3390/microorganisms14102351 (PMC13707883; doi:10.3390/microorganisms14102351)
Supplement: Supplementary file 1 [file microorganisms-14-02351-s001.zip › microorganisms-4568672-supplementary.pdf]

Supplementary Table S1 Resistance phenotypes of clinical M. tuberculosis isolates.

Strain	phenotype					Resistance category
	RIF	INH	MFX	BDQ	CFZ	
H37Rv	S	S	S	S	S	susceptible
39621[b]	S	S	S	S	S	susceptible
39614[b]	S	S	S	S	S	susceptible
39661[b]	S	S	R	I	S	MFX-resistant
39655[b]	S	S	S	I	S	susceptible
39773[b]	S	R	S	R	R	INH、BDQ、CFZ-resistant,
39737[b]	S	R	S	S	S	INH-resistant
39751[b]	S	R	S	S	S	INH-resistant
39779[b]	S	R	S	S	S	INH-resistant
39785[b]	S	R	S	S	S	INH-resistant
39600[b]	S	R	S	I	I	INH-resistant
39682[b]	S	R	S	I	I	INH-resistant
39719[b]	S	R	S	S	S	INH-resistant
39545[b]	S	R	S	S	S	INH-resistant
152[b]	R	S	S	S	S	RIF-resistant
39749[b]	R	R	S	S	S	Multidrug-resistant
39500[b]	R	R	S	S	S	Multidrug-resistant
39595[b]	R	R	R	S	S	Pre-extensively drug-resistant
39613[b]	R	R	I	S	S	Multidrug-resistant
39563[b]	R	R	R	S	S	Pre-extensively drug-resistant
11[b]	I	R	S	S	S	Multidrug-resistant
95[b]	R	R	S	S	S	Multidrug-resistant
27[b]	R	R	R	S	S	Pre-extensively drug-resistant

Note: S, susceptible; I, intermediate; R, resistant. MICs were determined by 7H9 broth microdilution. Interpretive criteria for 7H9 BMD: INH ($S \leq 0.12$, $R \geq 0.25$ μg/mL), RIF ($S \leq 1.0$, $R \geq 2.0$ μg/mL), MFX ($S \leq 0.25$, $I = 0.5$, $R \geq 1.0$ μg/mL), BDQ

(S ≤ 0.125, I = 0.25, R ≥ 0.5 μg/mL), CFZ (S ≤ 0.5, I = 1.0, R ≥ 2.0 μg/mL). Multidrug-resistant denotes resistance to at least isoniazid and rifampicin. Pre-extensively drug-resistant denotes multidrug-resistant plus resistance to any fluoroquinolone.
